# Visualization of multimodal polymer-shelled contrast agents using ultrasound contrast sequences: an experimental study in a tissue mimicking flow phantom

**DOI:** 10.1186/1476-7120-11-33

**Published:** 2013-08-29

**Authors:** Malin Larsson, Matilda Larsson, Letizia Oddo, Silvia Margheritelli, Gaio Paradossi, Jacek Nowak, Lars-Åke Brodin, Kenneth Caidahl, Anna Bjällmark

**Affiliations:** 1 Department of Medical Engineering, School of Technology and Health, KTH Royal Institute of Technology, Alfred Nobels Allé 10, 141 52, Huddinge, Stockholm, Sweden; 2 Department of Molecular Medicine and Surgery, Karolinska Institutet, Solna, Sweden; 3 Department of Chemical Sciences and Technologies, Università di Roma Tor Vergata, Rome, Italy; 4 Division of Clinical Physiology, Karolinska University Hospital, Huddinge, Sweden

**Keywords:** Acoustic shadowing, Contrast agent, Contrast sequences, Contrast to tissue ratio, Flow phantom, Multimodal

## Abstract

**Background:**

A multimodal polymer-shelled contrast agent (CA) with target specific potential was recently developed and tested for its acoustic properties in a single element transducer setup. Since the developed polymeric CA has different chemical composition than the commercially available CAs, there is an interest to study its acoustic response when using clinical ultrasound systems. The aim of this study was therefore to investigate the acoustic response by studying the visualization capability and shadowing effect of three polymer-shelled CAs when using optimized sequences for contrast imaging.

**Methods:**

The acoustic response of three types of the multimodal CA was evaluated in a tissue mimicking flow phantom setup by measuring contrast to tissue ratio (CTR) and acoustic shadowing using five image sequences optimized for contrast imaging. The measurements were performed over a mechanical index (MI) range of 0.2-1.2 at three CA concentrations (10^6^, 10^5^, 10^4^ microbubbles/ml).

**Results:**

The CTR-values were found to vary with the applied contrast sequence, MI and CA. The highest CTR-values were obtained when a contrast sequence optimized for higher MI imaging was used. At a CA concentration of 10^6^ microbubbles/ml, acoustic shadowing was observed for all contrast sequences and CAs.

**Conclusions:**

The CAs showed the potential to enhance ultrasound images generated by available contrast sequences. A CA concentration of 10^6^ MBs/ml implies a non-linear relation between MB concentration and image intensity.

## Background

In 1968, intravenously injectable air microbubbles (MBs) were introduced as a contrast agent (CA) for use with echocardiography in order to enhance echo from relatively weak echogenic regions such as the vascular lumen [1]. However, the instability of these free gas MBs with a lifetime of only a few seconds limited their practical use and their relatively large size hindered their passage through the pulmonary capillary. In order to improve the MB stability, attempts have been made to encapsulate gas within a thin shell and in 1984, the first MBs with ability to pass through the pulmonary circulation were developed [2]. However, the thin shell of these MBs still allowed air to diffuse across the shell structure. In order to optimize the MB stability, a low soluble gas, instead of air, was encapsulated within the shell and the gas leakage was thus limited. At present, CAs consisting of MBs with the improved stability are widely used during standard ultrasound examinations, enabling e.g. tissue perfusion studies and endocardial border delineation [3,4].

Although the commercially available CAs are relatively stable and well functioning in different clinical applications, there is still a need for improvement and extended applicability, such as targeted imaging and fusion imaging. In fact, polymer-shelled CAs with multimodality potential are currently under development [5,6]. The multimodality approach has the potential to increase the clinical value of contrast imaging by providing the possibility of retrieving anatomical and functional information from two or more imaging systems simultaneously. In addition, the attachment of specific antibodies and ligands to the shell surface could enable specific attraction of the MBs to intended targets. The CAs might also be employed as a carrier of drugs that could be released locally at chosen target sites by disruption of the MBs with high-energy ultrasound. Other advantages of these CAs are a long shelf life as well as a narrow size distribution that increases the image sensitivity, which is of primary importance during targeting imaging [7-9].

Nevertheless, it is important to remember that polymer-shelled MBs demonstrate a different acoustical behavior than lipid-shelled MBs due to differences in compressibility and visco-elastic properties. The acoustic properties of the polymeric CAs has so far been studied in-vitro using single element transducers (Poehlmann et al.: On the interplay of structural, mechanical and acoustic behaviour of multifunctional magnetic microbubbles, submitted) [10,11] and by using a commercially ultrasound scanner with in-house developed contrast sequences [12,13]. However, no in-vitro studies using available clinical ultrasound systems with optimized sequences for contrast imaging has been performed hitherto. Keeping this in mind, the aim of this study was to determine the acoustic response by studying the visualization capability of three polymer-shelled CAs – one plain ultrasound and two surface-modified MBs for combined ultrasound and magnetic resonance imaging - in a tissue mimicking flow phantom using different ultrasound contrast sequences. In addition, the acoustic shadowing at different concentrations of the developed CAs was studied.

## Material and methods

**Figure 1:**
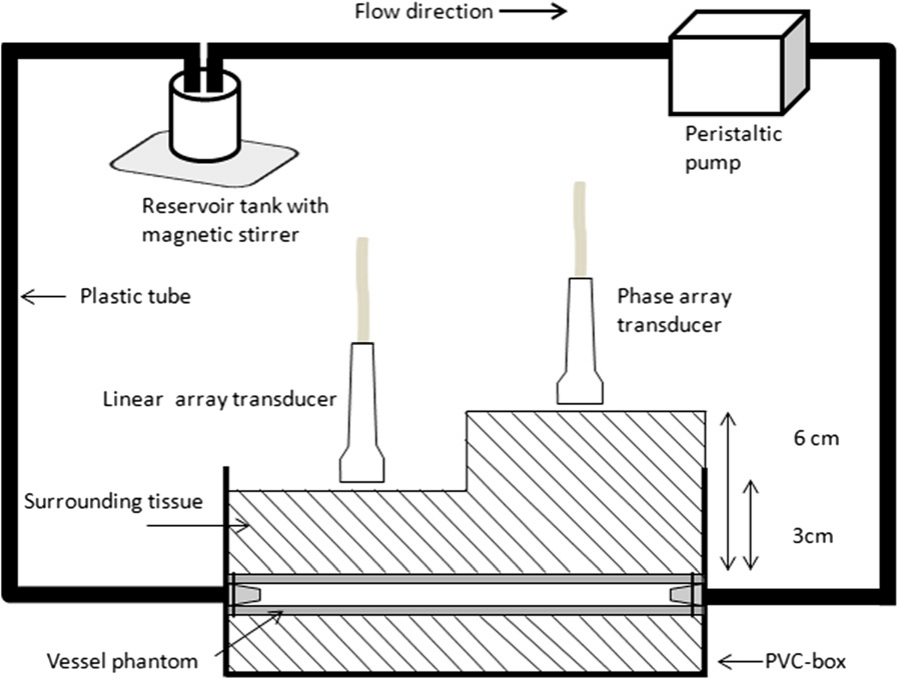
**Schematic illustration of the experimental setup.** A tissue mimicking flow phantom fixed in a polyvinyl chloride (PVC) box. The transducers were placed at two different depths relative to the vessel phantom lumen (3 cm, 6 cm) depending on the center frequency.

An experimental setup including a tissue mimicking flow phantom, a reservoir tank and a peristaltic pump was designed as shown in Figure 1. The contrast to tissue ratio (CTR) and the acoustic shadowing were studied for three polymer-shelled CAs, using different ultrasound systems with associated contrast sequences. The tests were performed at three CA concentrations. For the CTR-measurements, the commercially available CA SonoVue® was used as reference.

### Phantom design

**Figure 2:**
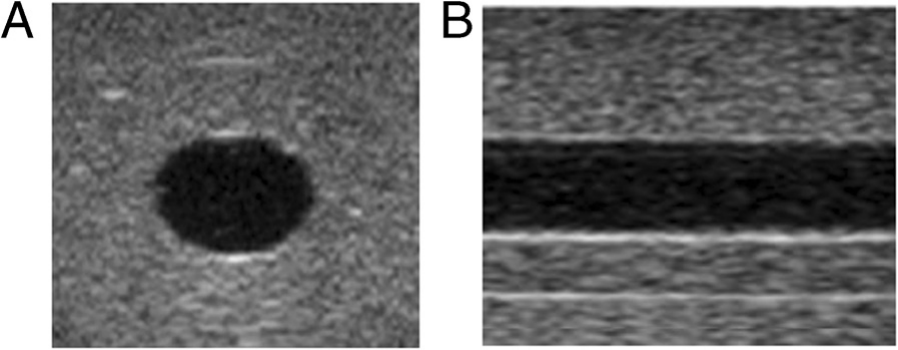
**Ultrasound B-mode images of the flow phantom.** Images acquired in the short axis **(A)** and the long axis view **(B)**. Circulated solution consisted of de-ionized water.

A tissue mimicking flow phantom with a vessel size of the human carotid artery was designed, see Figure 2. The vessel phantom was constructed by heating a mixture of 15% (w/w) polyvinyl alcohol (PVA) (Sigma-Aldrich, St. Louise, MO, US), 3% (w/w) graphite powder (particle size < 50 μm, Merck KGaA, Darmstadt, Germany) and de-ionized water to 90°C. Graphite powder was added as acoustic scatters [14]. The mixture was then poured into a cylindrical vessel mould of acrylic plastic (diameter 12 mm, length 100 mm) and the vessel lumen was created by inserting a metal rod (diameter 6 mm) into the vessel mould. Thereafter, it was stored in a freezer at −20°C for 12 h. During the subsequent 12 h, the vessel mould was stored in room temperature. In order to obtain acoustical properties similar to tissue, the vessel phantom underwent three freeze-thaw cycles [15].

After the three freeze-thaw cycles, the vessel phantom was attached to plastic connectors on each side of a polyvinyl chloride (PVC) box (100 mm × 60 mm), see Figure 1. The inner part of the PVC-box was covered with a 4 mm thick rubber layer that acted as an ultrasound absorbing material. A surrounding tissue mimicking mixture of 3% (w/w) agar (Merck KGaA, Darmstadt, Germany), 4% (w/w) graphite powder (particle size < 50 μm, Merck KGaA, Darmstadt, Germany) and de-ionized water was heated to 85°C. To prevent the vessel phantom from dissolving, the tissue mimicking mixture was cooled in room temperature to approximately 50°C before pouring it into the PVC-box. In addition, the vessel phantom lumen was filled with de-ionized water to prevent it from collapsing when pouring the tissue mimicking mixture into the PVC-box.

### Contrast agents

Three in-house made polymer-shelled CAs were employed in the study (Table 1). One type was a plain polymer-shelled CA (Plain PVA) useful solely with ultrasound imaging [6]. The other two types had a modified shell or shell surface, both containing superparamagnetic iron oxide nanoparticles (SPIONs) for combined ultrasound and magnetic resonance imaging. However, the binding technique of the SPIONs differed. In the modified CA Type A (PVA Type A), the SPIONs were covalently linked to the shell surface through chitosan molecules, whereas in the modified CA Type B (PVA Type B), the SPIONs were embedded in the shell [5,16]. In addition, the commercially available lipid-shelled CA SonoVue® was used as a reference [17].

**Table 1:** Properties of the contrast agents

**Type of CA**	**Shell material**	**Gas composition**
Plain PVA	Polymer	Air
PVA Type A	Polymer + SPION	Air
PVA Type B	Polymer + SPION	Air
SonoVue®	Phospholipid	Sulfur hexafluoride

PVA = polyvinyl alcohol, CA = contrast agent, SPION = superparamagnetic iron oxide nanoparticles.

The stock solution concentrations of each CA were determined using a light microscope (Olympus, Hamburg, Germany) and a counting chamber (Marienfeld Superior, Lauda-Königshofen, Germany). The mean value of the number of MBs within 16 squares of the chamber, each with a volume of 6.25 × 10^-3^ mm^3^, was calculated and displayed as the stock solution concentration. Subsequently, each stock solution was diluted with de-ionized water to desired concentration. In total, three concentrations of each type of CA were prepared, i. e. 10^4^, 10^5^ and 10^6^ MBs/ml.

### Experimental procedure

The tissue mimicking flow phantom was connected to a peristaltic pump (Watson Marlow, Falmouth, United Kingdom) generating a pulsatile flow with a mean velocity of 8.8 cm/s. The 500 ml solution circulating in the closed system consisted of de-ionized water and CA, which underwent constant stirring in the reservoir tank to obtain an even distribution of CA in the solution. A cleaning procedure was performed after each experiment with a specific CA at a given concentration in order to remove all MBs in the closed system. During the cleaning procedure, the vessel phantom and the plastic tubes were washed with de-ionized water. In total, three cleaning procedures were performed before a new batch of CA was added to the system.

The employed ultrasound transducers were fixed by a tripod holder at two different distances from the vessel phantom lumen depending on the applied frequency. The low frequency transducers (M3S (GE Healthcare, Wisconsin, US) and S5-1 (Philips Healthcare, Amsterdam, The Netherlands)) were fixed at a distance of 6 cm from the vessel lumen while the high frequency transducers (M12L (GE Healthcare, Wisconsin, US) and 9 L4 (Siemens Healthcare, Erlangen, Germany)) were fixed 3 cm from the vessel lumen.

### Contrast sequences

Four commonly used contrast sequences were employed in the study; pulse inversion (PI), power modulation (PM), contrast pulse sequence (CPS) and power pulse inversion (PPI) (Table 2). The common feature for all contrast sequences used, was the detection of the nonlinear response from the studied CA upon exposure to acoustic pressure. A brief description of each contrast sequences follows. In PI every second transmitted pulse was inverted compared to the previous, while in PM two pulses were transmitted in phase but with amplitude modifications [18]. For CPS a combination of both phase and amplitude modification was applied [19]. When applying PPI, a sequence of three pulses was transmitted for each echo line. The first and the third transmitted pulses are identical while the second transmitted pulse is an inverted replica of them [20].

**Table 2:** Characteristics and settings of the contrast sequences

**Ultrasound system**	**Contrast sequence**	**Transducer**	**Frequency (MHz)**	**Frame rate (Hz)**	**MI-range**
Vivid7, GE	PI	M12L	5/10	32.7	0.2-1.2
Vivid7, GE	PI	M3S	1.5/3.1	25.7	0.2-0.8
iE33, Philips	PM	S5-1	1.5/3.2	39	0.2-1.2
iE33, Philips	PPI	S5-1	1.3/2.6	39	0.2-1.0
Acuson sequoia 512, Siemens	CPS	9 L4	4/8	17	0.2-1.0

PI = pulse inversion, PM = power modulation, PPI = power pulse inversion, CPS = contrast pulse subtraction, MI = mechanical index.

### Data acquisition

Ultrasound short-axis images (n = 38) were acquired for four different contrast sequences (PI, PM, PPI and CPS) and three concentrations for each CA at a mechanical index (MI) ranging from 0.2 to maximal MI-value (0.8-1.2) of each ultrasound system tested (Table 2). A wide MI-range was used since the nonlinear responses from the CAs vary with applied acoustic pressure and the visco-elastic properties of the CA [21]. Before image acquisition, the focus point was placed in the middle of the vessel phantom lumen. The order of the image acquisition with the different contrast sequences was determined by a random number generator in Matlab® (MathWorks, Natick, US) to minimize the influence of possible concentration decrease of the CAs due to ultrasound exposure.

### Data analysis

All the image sequences were analyzed offline using a specific work station for each ultrasound system; EchoPAC (GE Healthcare, Wisconsin, US), QLAB (Philips Healthcare Amsterdam, The Netherlands), Syngo (Siemens Healthcare, Erlangen, Germany).

### Contrast to tissue ratio

**Figure 3:**
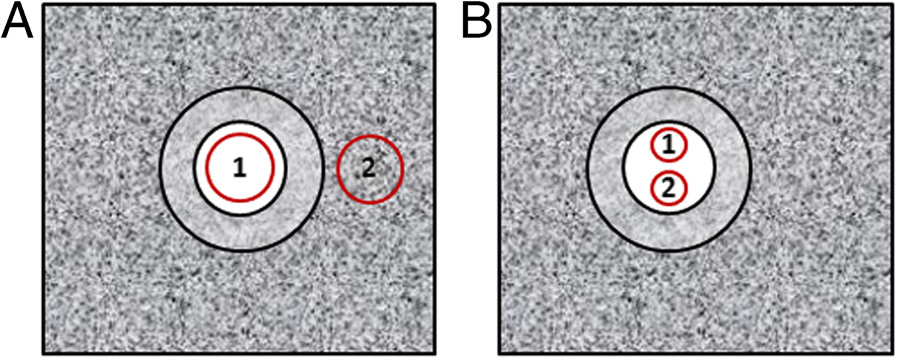
**Off-line measurements. A)** Illustration of the contrast to tissue ratio (CTR) measurements in the short axis view of the flow phantom. Two identical regions of interest (ROIs) were placed at the same depth in the short axis view; ROI_Vessel_ (1) and ROI_Tissue_ (2), **B)** Illustration of the acoustic shadowing measurements with two identical ROIs placed underneath each other in the phantom vessel lumen; ROI_upper_(1) and ROI_lower_ (2).

Two regions of interest (ROIs) with an area of approximately 12.5 mm^2^ were manually placed in the middle of the vessel phantom lumen (ROI_Vessel_) and in the surrounding tissue surrogate (ROI_Tissue_) at the same depth for all stored imaging sequences (Figure 3A). The ratio between the mean intensity (I), measured in decibel (dB), within the ROI_Vessel_ and the ROI_Tissue_ was calculated and displayed as CTR (Equation 1).

(1) $$\mathrm{CTR}=I_{\mathrm{RO}I_{\text{Vessel}}}-I_{\mathrm{RO}I_{\text{Tissue}}}$$

### Acoustic shadowing

In order to assess the acoustic shadowing, two identical ROIs, with an area of approximately 3 mm^2^, were manually placed in the upper and the lower part of the vessel phantom lumen (Figure 3B) [22]. A shadowing effect was assumed if a significant higher intensity was observed for the upper ROI compared with the lower ROI. This was tested for each contrast sequence and CA concentration at the MI at which the maximal CTR-value was obtained using one-sided paired t-test (confidence interval of 95%).

## Results

### Contrast to tissue ratio

**Figure 4:**
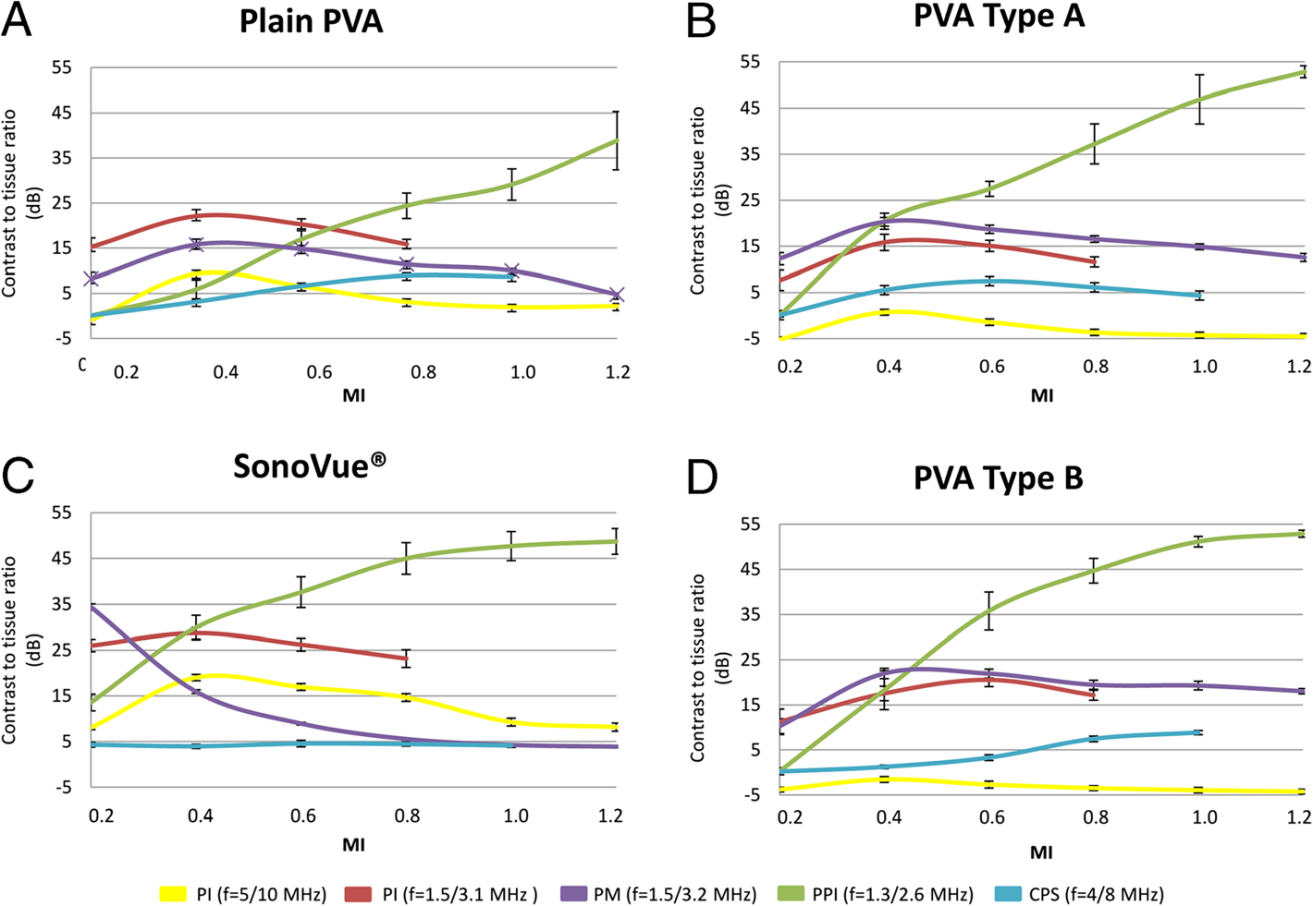
**Contrast to tissue ratio as a function of mechanical index (MI).** Contrast to tissue ratio (dB) (n = 38) as a function of MI for the different contrast agents (CAs) (Plain polyvinyl alcohol (PVA) **(A)**; PVA Type A **(B)**; PVA Type B **(C)**; SonoVue® **(D)**) and contrast sequences (pulse inversion, f = 5/10 MHz (yellow); pulse inversion, f = 1.5/3.1 MHz (red); power modulation, f = 1.5/3.2 MHz (purple); power pulse inversion, f = 1.3/2.6 MHz (green); contrast pulse sequence, f = 4/8 MHz (blue) included in the study. The concentration of the CAs was 10^5^ microbubbles/ml.

**Figure 5:**
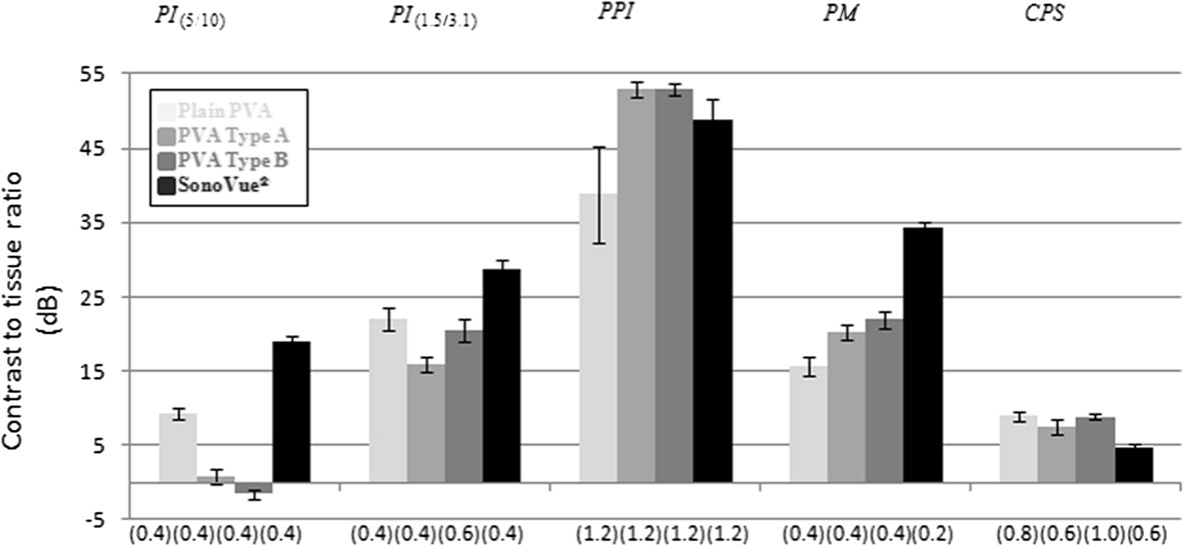
**Maximal contrast to tissue ratio.** The maximal contrast to tissue ratio (dB) value (n = 38) and its associated mechanical index (brackets) for each contrast agents (Plain PVA (light gray), PVA Type A (gray), PVA Type B (beige) and SonoVue® (black)) observed with the different contrast sequences (PI=pulse inversion, PM=power modulation, PPI=power pulse inversion, CPS=contrast pulse sequence). Negative CTR-values indicate that the tissue response exceeded the response from the CA. The concentration of the CAs was 10^5^ microbubbles/ml.

The distribution of the CTR-values expressed as a function of MI for different CAs at a concentration of 10^5^ MBs/ml obtained with different contrast sequences are presented in Figure 4. As can be seen, using the PI and PM sequences, the highest CTR-values were obtained at a lower MI (0.4), whereas with PPI sequence, the highest CTR-values appeared at higher MI (1.2). On the other hand, CPS sequence tended to produce the highest CTR-values at intermediate to higher MI (0.6 – 1.0). A similar distribution pattern of the CTR-values was found for the two other CA concentrations (10^4^ and 10^6^ MBs/ml) that were also tested (data not shown). This is further elucidated in Figure 5 where it shows the maximum CTR-values for each studied CA at a concentration of 10^5^ MBs/ml and the corresponding MI obtained with each contrast sequence employed. As can be seen from the figure, the PPI sequence provides the overall best performance, with the highest CTR-values for all studied CAs. A high dependency of the highest CTR values produced with different contrast sequences on the applied MI was also apparent in this study, a higher MI being optimal for use with PPI and CPS and a lower MI optimal for use with PI and PM sequences. Furthermore, PI and PM provided higher CTR-values for SonoVue® compared with the polymer-shelled CAs.

**Figure 6:**
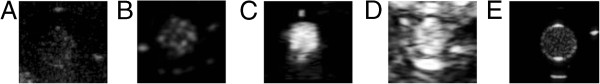
**Ultrasound images of the tissue mimicking flow phantom containing Plain PVA.** The images were obtained at maximum contrast to tissue ratio for each contrast sequences: **A)** Pulse inversion (f = 5/10 MHz, MI = 0.4), **B)** Pulse inversion (f = 1.5/3.1 MHz, MI = 0.4), **C)** Power pulse inversion (f = 1.3/2.6 MHz, MI = 1.2), **D)** Power modulation (f = 1.5/3.2 MHz, MI = 0.4), **E)** Contrast pulse sequence (f = 4 MHZ, MI = 1.0). The concentration of the CAs was 10^5^ microbubbles/ml.

Figure 6 shows examples of ultrasound images of the tissue mimicking flow phantom containing Plain PVA at a concentration of 10^5^ MBs/ml obtained with different contrast sequences at maximum CTR-values. The figure illustrates in further detail the high CTR produced by PPI sequence (Figure 6C) as well as a clear frequency-dependent difference in the images generated by PI with a high frequency transducer (f = 5/10 MHz) and low frequency transducer (f = 1.5/3.1 MHz), the higher CTR-values being obtained with the low frequency transducer (compare Figure 6A and 6B).

### Acoustic shadowing

At a concentration of 10^6^ MBs/ml, significant acoustic shadowing was observed for all three polymer-shelled CAs and contrast sequences (see Table 3). For concentrations lower than 10^5^ MBs/ml, significant acoustic shadowing was only observed when using PPI.

**Table 3:** Acoustic shadowing

**CA**	**PI**			**PI**			**PM**			**PPI**			**CPS**		
	**(f = 5/10 MHZ)**			**(f = 1.5/3.1 MHz)**			**(f = 1.5/3.2 MHz)**			**(f = 1.3/2.6 MHz)**			**(f = 4 MHz)**		
	**10**^**6**^	**10**^**5**^	**10**^**4**^	**10**^**6**^	**10**^**5**^	**10**^**4**^	**10**^**6**^	**10**^**5**^	**10**^**4**^	**10**^**6**^	**10**^**5**^	**10**^**4**^	**10**^**6**^	**10**^**5**^	**10**^**4**^
Plain PVA	p < 0.001	NS	NS	p < 0.001	NS	NS	p < 0.001	NS	NS	p < 0.001	p < 0.01	NS	p < 0.001	NS	NS
	(0.4)	(0.4)	(0.4)	(0.4)	(0.4)	(0.4)	(0.4)	(0.4)	(0.4)	(1.2)	(1.2)	(1.2)	(1.0)	(0.8)	(0.8)
PVA Type A	p < 0.001	NS	NS	p < 0.001	p < 0.001	NS	p < 0.001	p < 0.001	NS	p < 0.001	p < 0.001	p < 0.001	p < 0.001	NS	NS
	(0.4)	(0.4)	(0.4)	(0.4)	(0.4)	(0.4)	(0.4)	(0.4)	(0.6)	(1.2)	(1.2)	(1.2)	(0.8)	(0.6)	(0.6)
PVA Type B	p < 0.001	NS	NS	p < 0.001	NS	NS	P = 0.01	NS	NS	p < 0.001	p < 0.001	p < 0.05	p < 0.001	NS	NS
	(0.4)	(0.4)	(0.4)	(0.4)	(0.6)	(0.4)	(0.6)	(0.4)	(0.6)	(1.2)	(1.2)	(1.2)	(1.0)	(1.0)	(1.0)

Observations of acoustic shadowing, at the maximal contrast to tissue ratio (dB) value and its associated mechanical index (brackets) for the different types of the polymer-shelled shelled CA at three concentrations; 10^6^ MBs/ml, 10^5^ MBs/ml, 10^4^ MBs/ml. A significant shadowing effect is marked with its p-value and a non significant shadowing effect with NS (not significant).

PI = Pulse Inversion, PM = Power modulation, PPI = power pulse inversion, CPS = Contrast pulse sequence, PVA = Polyvinyl alcohol, NS = not significant.

## Discussion

The CTR-values from the different CAs was strongly dependent on the contrast sequence and MI applied. As in previous studies [5,10,13] (Poehlmann et al.: On the interplay of structural, mechanical and acoustic behaviour of multifunctional magnetic microbubbles, submitted), variations in acoustic response for the polymer-shelled CAs were observed, thus suggesting that surface modification has an impact on the acoustic properties.

Overall, the PPI generated the highest CTR-values for all CAs included in the study. PPI is a decorrelation detection contrast sequence with the ability to effectively cancel the fundamental signal and enhance the harmonic signals due to the transmission and detection of three pulses for each echo line [20]. Both the oscillation and the destruction of CAs between the pulses, results in a high degree dissimilarities of the harmonic components, especially at high pressure, i.e. at higher MI. However, not only CA, but tissue also generates a nonlinear response when exposed to higher MI. Therefore, in order to obtain an image with minimal tissue influence, the received signal is filtered between the first and the second harmonic peak for the PPI contrast sequence. The main reason for this is that the harmonic signals from the CA have a wider bandwidth than those from the surrounding tissues [18]. The use of flexible and easily destroyed CA would result in a high decorrelation of the returning ultrasound pulses, which appears to be optimal for PPI sequence. This fact is confirmed by the high CTR-values for the thin-shelled SonoVue® using PPI. However, the thick polymer-shelled CAs used in this study produced CTR-values of the same magnitude as SonoVue®, even though the thick-shelled CAs fracture at higher MIs and this process is different compared to thin-shelled MBs [11]. The strong acoustic signal from the polymer-shelled CAs using PPI may be a result of shell defect caused by the ultrasound exposure resulting in release of free gas [18].

The ability to generate harmonics is highly dependent on the visco-elastic properties of the shell, which are determined by the shell thickness and its composition [21]. The polymer-shelled CAs have a stiffer and thicker shell than SonoVue®, implying that a higher MI is needed to obtain a sufficient nonlinear response from these CAs [23,24]. But, in the case for PM and PI, a higher MI generates too much non-linear tissue signal to obtain high CTR-values. The difference in shell properties appears therefore to be the main reason for the fact that PI and PM perform better with SonoVue® than with the polymeric shelled CAs.

The two tested contrast sequences based on PI (low frequency f = 1.5/3.1 MHz and high frequency f = 5/10 MHz) generated different acoustic response, the lower frequency being superior for all CAs tested. The flexible shell of SonoVue® allows for large radius excursions giving rise to a well-defined resonance frequency [25]. This resonance frequency corresponds to the frequency range of the low frequency transducer, which in fact explains the higher CTR-values in this case. The polymer-shelled CAs are stiffer, which results in a considerable damping and a higher and less well-defined resonance frequency. Nevertheless, the low frequency transducer generated the highest CTR-values also with these CAs. One probable explanation to the observed phenomena, is that the high frequency signal results in higher pressure amplitudes at a certain MI, which cause a higher nonlinear response from tissue and hence, a decreased CTR-value. The frequency-dependant progressive distortion of the wave as it travels distally is an additional explanation. This influence was, however, reduced in the present experimental setup by the different imaging depths for the low and high frequency transducers.

The penetration of the ultrasound beam at a high concentration of MBs is reduced due to scattering and absorption [26]. It has been shown that acoustic shadowing is less pronounced at concentrations where there is a linear relationship between the concentration of CA and backscattered intensity [27]. In the present study, acoustic shadowing was observed for concentrations of 10^6^ MBs/ml, irrespective of the CA and contrast sequence used, indicating a non-linear relation between CA concentration and image intensity at this concentration. Acoustic shadowing was most pronounced for PPI, where significant shadowing was observed even at low CA concentrations. This can, at least partly, be explained by the high pressure amplitudes for this contrast sequence, which in other studies have shown to highly increase the attenuation in a contrast media suspension [28-30]. These findings are contradictory to the observation for CPS, where acoustic shadowing was not observed for the lower CA concentrations of the polymeric CA even though the pressure amplitude and the frequency was higher than for PPI. However, it should be mentioned in this context that the attenuation also has shown to be dependent on the transducer design and incident frequency spectra [28]. These results were found in a single element transducer setting but related findings was also obtained in a study utilizing clinical ultrasound systems [31]. In that study, machine-specific pre-and post processing algorithms were pointed out as an explanation for variation in the relationship between CA concentration and video intensity between ultrasound systems. In the current performed study, broadband transducers were used and the impact of the transducer design in combination with other machine-specific factors remains to be defined.

The in-vitro flow phantom setup offers standardized conditions for image acquisition. However, some limitations must be taken into account. It would have been optimal to use one single ultrasound system equipped with different contrast sequences over a wide frequency range. This was not possible since the clinical ultrasound systems available on the market today are limited to a few specific frequencies and contrast sequences. Still, this study provides an indication of which settings to use in order to visualize the polymeric CAs. A closed system was employed in the present study, implying that concentration loss due to MB destruction might have influenced the obtained CTR-values. However, the short ultrasound exposure time in combination with a relatively large volume of the CA solution, limited the effect of any MB destruction. Furthermore, the image acquisition was also performed in a randomized order in order to minimize possible influence of MB destruction. Different frame rates were used in the study. As a consequence, the acquisition time differed between the ultrasound systems tested. However, variations within the stored image sequences were limited as a homogenous CA mixture was used. In addition, a relatively high flow was applied which minimize the effect of eventual MB destruction in the field of view. Furthermore, if air bubbles were observed, a new image sequence was obtained. MBs motion is known to influence multi-pulse techniques using phase modulations. Although it has been demonstrated that the influence of axial motion of the MBs is much higher than that of lateral motion [32], the lateral motion of the MBs in our experimental setting might have influenced the tested contrast sequences. The present experimental setup mimicked the in-vivo imaging situation in the macrocirculation, in which contrast enhanced imaging can be used for detection of various clinical scenarios, e.g. left ventricle opacification, endocardial border delineation and detection of atherosclerotic plaques. Nevertheless, in order to fully evaluate the potential of the polymer-shelled CAs, future studies need to focus on the ability to visualize the microcirculation and to quantify tissue perfusion.

The polymer-shelled CAs has high mechanical and chemical stability [8], facilitating a possible use of the MBs as a carrier for different substances that can be incorporated into the shell or attached to the shell surface. It has thereby the potential to be of value in new clinical applications as, for example, multimodality imaging, targeted imaging and drug delivery. In particular, multimodal imaging where ultrasound and MRI are combined can add incremental value since these imaging methods are complementary in the diagnostic practice. The results of this study provide a clear indication of the optimal settings and contrast sequences for the polymer-shelled CAs, however, in-vivo imaging will certainly be more challenging and factors such as injection speed and concentration focus position and gain levels remain to be optimized. There is therefore a need for new in-vitro and in-vivo studies in order to further evaluate the applicability of the developed CAs, especially in the evaluation of microcirculation and tissue perfusion.

## Conclusions

The present results demonstrate that the three polymer-shelled CAs have the potential to enhance ultrasound images when using commercially available contrast sequences, the highest CTR-values being obtained with PPI at higher MI. Surface modification seems to have an impact on the acoustic properties, as illustrated by the differences in CTR-values and acoustic shadowing between the three polymer-shelled CAs currently tested. A significant acoustical shadowing observed with all contrast sequences at a CA concentration of 10^6^ MBs/ml implies that this CA concentration is too high for a linear relation between MB concentration and image intensity.

## Competing interests

The authors declare that they have no competing interests.

## Authors’ contributions

ML, LÅB, AB participated in the initiation and design of the study. LO, SM and GP produced the polymer-shelled contrast agent used in the study. ML, ML, JN, KC and AB participated in data collection and analysis of the results. All authors read and approved the final manuscript.
